# Increase in choroidal thickness after blue light stimulation of the blind spot in young adults

**DOI:** 10.1186/s42234-024-00146-5

**Published:** 2024-06-03

**Authors:** Hosein Hoseini-Yazdi, Scott A. Read, Michael J. Collins, Hamed Bahmani, Jens Ellrich, Tim Schilling

**Affiliations:** 1 Contact Lens and Visual Optics Laboratory, Centre for Vision and Eye Research, Optometry and Vision Science, Queensland University of Technology, Brisbane, 4059 Australia; 2 Dopavision GmbH, Krausenstr. 9-10, 10117 Berlin, Germany; 3 Medical Faculty, Friedrich-Alexander-University Erlangen-Nuremberg, 91054 Erlangen, Germany

**Keywords:** Melanopsin, Intrinsically photosensitive retinal ganglion cell, Optic disc, Axial length, Myopia

## Abstract

**Background:**

Blue light activates melanopsin, a photopigment that is expressed in intrinsically photosensitive retinal ganglion cells (ipRGCs). The axons of ipRGCs converge on the optic disc, which corresponds to the physiological blind spot in the visual field. Thus, a blue light stimulus aligned with the blind spot captures the ipRGCs axons at the optic disc. This study examined the potential changes in choroidal thickness and axial length associated with blue light stimulation of melanopsin-expressing ipRGCs at the blind spot. It was hypothesized that blue light stimulation at the blind spot in adults increases choroidal thickness.

**Methods:**

The blind spots of both eyes of 10 emmetropes and 10 myopes, with a mean age of 28 ± 6 years (SD), were stimulated locally for 1-minute with blue flickering light with a 460 nm peak wavelength. Measurements of choroidal thickness and axial length were collected from the left eye before stimulation and over a 60-minute poststimulation period. At a similar time of day, choroidal thickness and axial length were measured under sham control condition in all participants, while a subset of 3 emmetropes and 3 myopes were measured after 1-minute of red flickering light stimulation of the blind spot with a peak wavelength of 620 nm. Linear mixed model analyses were performed to examine the light-induced changes in choroidal thickness and axial length over time and between refractive groups.

**Results:**

Compared with sham control (2 ± 1 μm, *n* = 20) and red light (−1 ± 2 μm, *n* = 6) stimulation, subfoveal choroidal thickness increased within 60 min after blue light stimulation of the blind spot (7 ± 1 μm, *n* = 20; main effect of light, *p* < 0.001). Significant choroidal thickening after blue light stimulation occurred in emmetropes (10 ± 2 μm, *p* < 0.001) but not in myopes (4 ± 2 μm, *p* > 0.05). Choroidal thickening after blue light stimulation was greater in the fovea, diminishing in the parafoveal and perifoveal regions. There was no significant main effect of light, or light by refractive error interaction on the axial length after blind spot stimulation.

**Conclusions:**

These findings demonstrate that stimulating melanopsin-expressing axons of ipRGCs at the blind spot with blue light increases choroidal thickness in young adults. This has potential implications for regulating eye growth.

## Background

Among the modifiable risk factors associated with myopia, increasing outdoor time and the associated high light levels have received significant attention to reduce the risk of developing myopia. Increasing evidence suggests that high ambient light has a protective effect against excessive eye growth and myopia development, potentially through the retinal dopaminergic pathway (Norton and Siegwart 2013). Intrinsically photosensitive retinal ganglion cells (ipRGCs) are a small subset of retinal ganglion cells that express the photopigment melanopsin (Fu et al. 2005) and respond directly to blue light with a peak sensitivity of approximately 480 nm. These cells have slow response kinetics characterized by long latency and poststimulus persistence (Mure et al. 2009, 2019; Stone et al. 2013) and are implicated in light-mediated mechanisms regulating eye growth and myopia development. Melanopsin signaling has been shown to contribute to refractive growth of the eye in mice (Liu et al. 2022). A lack of intrinsic melanopsin signaling was associated with a myopic shift in refraction (Chakraborty et al. 2022a), whereas selective chemogenetic activation of melanopsin led to a hyperopic shift (Liu et al. 2022). ipRGCs communicate with dopaminergic amacrine cells in the retina (Newkirk et al. 2013; Norton and Siegwart 2013; Schmidt et al. 2014; Stone et al. 2013), and their activation is suggested to inhibit myopia development through a dopaminergic signaling cascade (Troilo et al. 2019; Witkovsky 2004). However, there is currently a lack of strong evidence supporting the role of ipRGCs in the light-dependent processes of eye growth in humans.

ipRGCs in both animals (Esquiva et al. 2016; Fahrenkrug et al. 2004; Hattar et al. 2002) and humans (Hannibal et al. 2017) express the photopigment melanopsin in their axons, as well as their cell bodies. The axons of the ipRGCs are bundled at the optic disc before projecting to the brain. Melanopsin-containing axons were observed in sections where the optic nerve exited the eye in rats (Esquiva et al. 2016). Axonal labeling was observed in the first 2 mm of the optic nerve in mice (Fahrenkrug et al. 2004). Recent studies support the ability to activate ipRGC-driven mechanisms via the blind spot by demonstrating changes in the contrast sensitivity (Schilling et al. 2023), the retinal electrical activity (Amorim-de-Sousa et al. 2021; Schilling et al. 2022), the pupillary light reflex (Miyamoto and Murakami 2015; Schilling et al. 2023), and the perception of brightness (Saito et al. 2018) in humans following blind spot stimulation with blue light. These findings suggest that selective stimulation of the blind spot with appropriate short wavelength stimuli can excite melanopsin, which is located on ipRGCs axons, and alter functional responses from the eye. The limitation of blue light stimulation to the blind spot provides selective stimulation of melanopsin because of missing cones and rods at this anatomical site (Güler et al. 2008). This stimulation approach aims at increasing choroidal thickness and decreasing axial length, both biomarkers of ocular growth regulation (Troilo et al. 2019).

There is compelling evidence for a relationship between choroidal thickness and vision-dependent processes that regulate the refractive state of the eye, with choroidal thinning associated with myopia and choroidal thickening associated with emmetropia or hyperopia (Nickla and Wallman 2010; Read et al. 2019; Wildsoet and Wallman 1995). Studies involving the current treatments for childhood myopia, including orthokeratology (Li et al. 2019; Wang et al. 2023), defocus incorporated multiple segments (DIMS) lenses (Chun et al. 2023), and atropine eye drops (Ye et al. 2020), suggest that short-term thickening of the choroid in response to these treatments predicts long-term slowing of eye growth and myopia progression. Recent studies on the human eye have also shown an increase in choroidal thickness in response to exposure to increased light levels (Chakraborty et al. 2022b), with evidence from animals further suggesting that melanopsin signaling contributes to the light-mediated increase in choroidal thickness independent of the rod pathway (Berkowitz et al. 2016). The present study tested the hypothesis that stimulating the melanopsin-expressing axons of the ipRGCs at the blind spot with blue light increases choroidal thickness in young adults.

## Methods

### Participants

Twenty young adults aged 18 to 35 years were enrolled in this study. This sample size provides 80% power to detect a significant change of ∼ 6 μm in the mean choroidal thickness, using a statistical significance level of 0.05 (Hoseini-Yazdi et al. 2019, 2020). The eligibility of participants was examined at a screening visit to include only those individuals who were in good general health, had normal best corrected vision (logMAR 0.00 or better in each eye), normal binocular vision, stable fixation by excluding nystagmus, and no history or evidence of amblyopia, strabismus, accommodation dysfunction, or any other significant ocular disease, ocular injury, or eye surgery. Smokers were not included. Participants using any administration of medication and those with a history of seizure, epilepsy, motion sickness, claustrophobia, adverse reactions to viewing flickering light, sleep disorders or poor sleep quality, determined via the Pittsburgh Sleep Quality Index questionnaire (Buysse et al. 1989), were excluded. Participants were also excluded if they were using treatments to control the progression of myopia, such as orthokeratology lenses, myopia control spectacles, soft contact lenses, or atropine eye drops, if they were using treatments to improve sleep quality, or if they were using blue blocking spectacles. Of the 22 participants who attended the screening visits, two participants did not meet the eligibility criteria (due to excessive astigmatism and poor sleep quality), resulting in the enrollment of 20 participants in the study. The Queensland University of Technology human research ethics committee approved the study. Written informed consent was obtained from each participant, and the study adhered to the tenets of the Declaration of Helsinki.

Based on non-cycloplegic subjective refraction of the left eye, participants were classified as emmetropes with a spherical equivalent refractive error (SER) ranging from − 0.25 Diopters (D) to + 0.75 D (*n* = 10) or as myopes with a SER ranging from − 0.50 D to −6.00 D (*n* = 10) (Flitcroft et al. 2019). Participants with astigmatic refractive errors greater than 1.50 D or anisometropia greater than 1.00 D were excluded from the study to limit any potentially confounding effects of uncorrected astigmatic defocus (Hoseini-Yazdi et al. 2020) or anisometropia (Vincent et al. 2013).

### Design

**Fig. 1:**
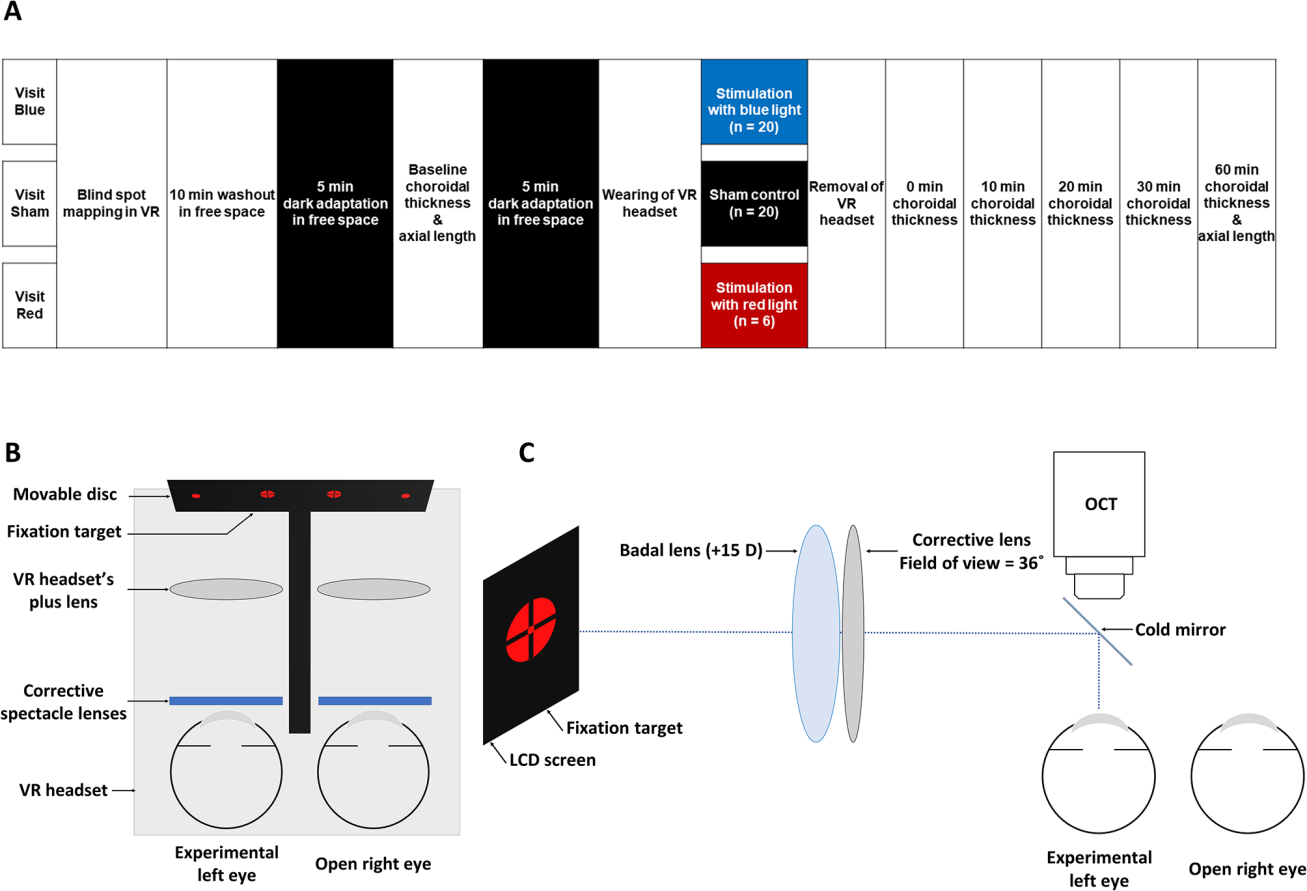
Overview of the experimental setup for stimulating the blind spot with blue light, sham control, and red light on separate days in a random order, indicated by visits Blue, Sham or Red respectively. Initially, blind spot mapping was performed in VR, followed by a 10 min washout in free space. A baseline OCT measurement was performed with 5 min of dark adaptation in free space before and after the procedure. After the 1-min stimulation of the blind spot in the VR headset, OCT measurements were performed after 0, 10, 20, 30, and 60 min. Optical biometry was performed at baseline and after 60 min (**A**). The position of the blind spot was determined through a calibration process for mapping the blind spot of each eye. A red disc was presented on a smartphone screen through a VR headset with 2.25 D accommodation demand, while any refractive error was optimally corrected with spectacle lenses (**B**). OCT images of the left eye were collected while the participants were viewing an external fixation target via a cold mirror and a Badal system to correct any refractive errors in the left eye during imaging (**C**). The axial length of the left eye was measured using optical biometry with a similar Badal system attached to the optical biometer

Eligible participants attended two experimental visits, during which the blind spot was stimulated with blue light or without any light stimulation as a sham control condition. A subset of participants, including 3 emmetropes and 3 myopes, was randomly selected from the original cohort and attended later an additional third experimental visit to stimulate with red instead of blue light as an active control (Fig. 1A). The experimental visits were conducted in a randomized order on different days at approximately the same time of day (12:48 ± 2:11 pm for the blue light condition, 12:16 ± 1:52 pm for the red light condition, and 12:44 ± 2:09 pm for the sham control condition) to control for potential diurnal effects on ipRGCs activity and choroidal thickness (Chakraborty et al. 2018). Participants refrained from consuming coffee or alcohol at least four hours prior to the commencement of each experimental visit.

### Procedure

#### Blind spot mapping

For mapping and light stimulation of the blind spot, a smartphone (Galaxy S7, Samsung, South Korea) viewed through a virtual reality (VR) headset (Merge VRG-01MG, Merge Labs, USA) was used (Fig. 1A).

At the beginning of each study visit, the volunteer was asked to determine the position of the blind spot for each eye in the VR environment by adjusting a small red disc with an angular size of ∼ 4° (smaller than the blind spot) in the peripheral visual field until it became completely invisible while fixating on a small central fixation target (Fig. 1B). The movable red disc used for blind spot mapping was created using a custom-written Android program (Dopavision GmbH, Germany). The position of the blind spot was saved by the program and used for subsequent stimulation. The accommodation demand was estimated to be 2.25 D during the VR experience for an eye optimally corrected for distance. To control for any confounding effect of accommodation, similar accommodation demand was present during the sham control, active control, and the blue light condition.

#### Washout

Blind spot mapping was followed by a 15-minute washout period in free space. This free space period involved viewing a grayscale movie for 10 min at distance with both eyes open and optimal sphero-cylindrical correction provided in a trial frame under ∼ 10 lx ambient lighting, followed by a 5-minute dark adaptation period with ambient lighting of < 1 lx. The initial washout period minimized any effects of prior near tasks (Hoseini-Yazdi et al. 2021) or light exposure (Lou and Ostrin 2020; Read et al. 2018) on the baseline choroidal thickness and axial length measurements. The 5-minute dark adaptation period before baseline optical coherence tomography (OCT) imaging also allowed for an adaptation response consistent with the dark adaptation before blind spot stimulation and subsequent OCT measurements, thus minimizing the potential confounding influence of darkness on choroidal thickness (Lou and Ostrin 2020) and the choroidal response to stimulation.

#### Baseline

Subsequent to washout, baseline measurements of choroidal thickness and axial length were carried out. During these measurements, optimal sphero-cylindrical correction of the left eye was provided using a Badal system attached to the OCT and optical biometer instruments (Hoseini-Yazdi et al. 2019, 2020), with a high-contrast bull’s eye plus cross-hair target (Thaler et al. 2013) used as the fixation target, consistent with the fixation target used in the VR headset (Fig. 1C). Participants viewed this external fixation target at optical infinity using a cold mirror, a + 15 D Badal lens and a corrective lens to correct for refractive error in the left eye while being imaged. The bright blue internal fixation target was switched off for the baseline and all subsequent OCT scans at both study visits to minimize any confounding effect of the Spectralis OCT instrument’s blue fixation target on the choroidal response to ipRGC stimulation. This procedure of turning off the instrument’s internal fixation light and using an external fixation target has been performed in previous studies (Hoseini-Yazdi et al. 2019, 2020).

#### Stimulation

Following baseline OCT imaging and optical biometry, both eyes were dark-adapted for 5 min (ambient lighting < 1 lx). The participant subsequently wore the VR headset and confirmed that the red calibration disc was still not visible when fixating on the central fixation target. The blind spot was then stimulated for 1 min with 12 Hz flickering blue light (peak wavelength 460 nm, luminance 22 cd/m^2^, angular size ∼ 4°), no light as a sham control, or flickering red light (peak 620 nm, luminance 139 cd/m^2^, and angular size ∼ 4°) as an active control condition, while the participant fixated on the central bull’s eye and cross-hair target.

The light stimulus was temporally modulated with a rectangular waveform at 12 Hz. Blue light stimulation at the blind spot with such flickering frequencies has been shown to reliably evoke pupil responses in a recent human experimental study (Adhikari et al. 2023). In deed, moderate flicker frequencies between 6 and 15 Hz tend to suppress experimentally induced myopia, increase dopamine synthesis in the retina (Kee et al. 2001; Rohrer et al. 1995; Schwahn and Schaeffel 1997), and lead to thickening of the choroid (Mathis et al. 2022).

None of the participants perceived the blue or red light stimulus in the periphery, although some perceived a marginal glow. This further confirmed the alignment of the light stimulus with the blind spot and stimulation of the blind spot. The ambient room light was maintained at 10 lx before and after the dark adaptation, and during blind spot stimulation throughout all the experimental sessions.

#### Measurements

Choroidal thickness was assessed at 0, 10, 20, 30, and 60 min following blind spot stimulation or sham control, using the same protocol as that used for the baseline measurements. Axial length was measured at 60 min poststimulation, immediately after the 60-minute OCT imaging. Between the measurement time points, the participants watched a grayscale movie, while the sphero-cylindrical refractive error of both eyes was optimally corrected in a trial frame. Therefore, any effects of optical defocus (Chakraborty et al. 2012; Chiang et al. 2015; Hoseini-Yazdi et al. 2019, 2020; Read et al. 2010) or chromatic cues (Lou and Ostrin 2020) from the screen on measures of choroidal thickness and axial length were minimized (Ostrin et al. 2023).

Eligible participants also underwent wide-field scanning laser ophthalmoscopy (SLO) fundus imaging that was captured in conjunction with wide-field volumetric horizontal OCT imaging during the screening visit using the Spectralis instrument to estimate the diameter of the blind spot corresponding to the optic disc. In this analysis, the termination of Bruch’s membrane was identified manually on each horizontal B-scan image intersecting the optic nerve, aligning with the optic disc margin on the SLO image. A best-fitting circle was then applied to the marked optic disc margin utilizing the graphical tools of the Heidelberg Explorer software. The measured diameter of the best-fitted circle was adjusted for the ocular magnification associated with ocular biometry and refraction to provide an estimate of the blind spot diameter in degrees.

### Instrumentation

Spectral domain optical coherence tomography (SD-OCT; Spectralis, Heidelberg, Germany) was used for choroid measurements, and optical biometry (Lenstar LS 900, Haag-Streit AG, Switzerland) was used for axial length measurements. Foveal-centered enhanced depth imaging OCT images were collected three times at baseline along the vertical meridian using a 30° high-resolution scan protocol, and 100 frames were averaged for each B-scan (Hoseini-Yazdi et al. 2018, 2019). The instrument’s follow-up mode was activated to ensure that all the B-scans collected at different measurement time points across the two visits were acquired from a location identical to the baseline scan of the first visit. Baseline optical biometry was always performed following the baseline OCT imaging, with five consecutive measurements collected.

### Data analysis

**Fig. 2:**
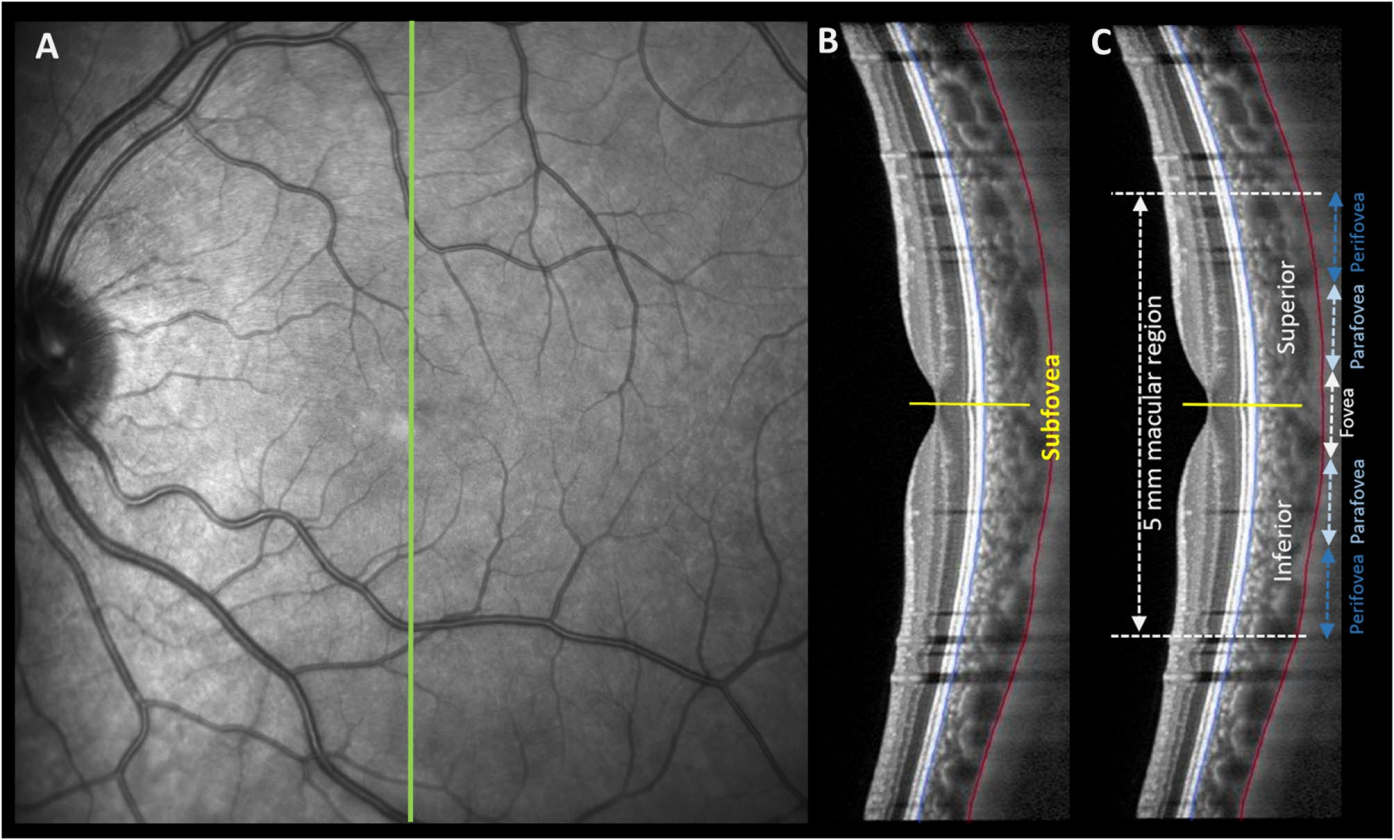
Illustration of the fundus en-face image and the position of the 30° OCT scan (green line in **A**) and the corresponding high-resolution enhanced depth imaging B-scan along the vertical meridian (**B** and **C**). Choroidal thickness was measured between the hyperreflective line corresponding to the outer surface of the retinal pigment epithelium and Bruch’s membrane complex (blue line in **B** and **C**) and the hyperreflective line corresponding to the inner surface of the choroidoscleral interface (red line in **B** and **C**) at the subfoveal point (i.e., the deepest point in the foveal pit shown with a yellow line in **B**) and across the fovea (central 1 mm region), parafovea (1–3 mm region adjacent to fovea), and perifovea (3–5 mm region adjacent to parafovea) in the same B-scan (**C**)

A custom-written program was used to automatically segment the anterior and posterior boundaries of the choroid (Kugelman et al. 2019), and segmentation inaccuracies were corrected by an experienced masked observer (Fig. 2A). The transverse magnification of each B-scan was then adjusted to account for variations in ocular refraction and biometry using a previously described method (Hoseini-Yazdi et al. 2019). This adjustment revealed a mean ± SD scan length of 9.05 ± 0.58 mm, ranging from 8.28 to 10.39 mm. Subsequently, the choroidal thickness was measured at the subfoveal point, which is defined as the deepest point of the foveal pit (Fig. 2B), and across the foveal, parafoveal, and perifoveal eccentricities over the macula, centered on the fovea (Fig. 2C). The fovea was the central 1 mm region, the parafovea was the 1–3 mm region next to the fovea, and the perifovea was the 3–5 mm region adjacent to parafovea. These regional measures of choroidal thickness were then averaged across the three repeated scans captured at each time point and used for analysis. The five repeated measurements of axial length were also averaged for measurements obtained at baseline and after 60 min of stimulation of the blind spot and were used for analysis.

#### Statistical analysis

All the statistical analyses were conducted using IBM SPSS Statistics 26 (www.ibm.com/software/analytics/spss). Given the different numbers of measurements collected across participants in the present study, a linear mixed model (LMM) analysis, which is capable of handling unbalanced data, was carried out (Cnaan et al. 1997). Separate LMM analyses were also conducted to examine the changes in subfoveal choroidal thickness, mean macular choroidal thickness, and axial length associated with blind spot stimulation with light. For subfoveal and mean macular choroidal thickness analyses, the main effects of light (three levels: sham, blue light, red light), time (five levels: 0, 10, 20, 30, 60 min) and refractive error (two levels: emmetropes, myopes) and the interactions between light, time, and refractive error were included as fixed factors. A repeated measure design was used in each LMM by including the slope and intercept of individual participants as random factors. A compound symmetry covariance structure was assumed for the repeated factors of light stimulation and time. For the axial length analysis, the main effects of light, refractive error, and the interaction of light by refractive error were included as fixed factors, and the slope and intercept of all participants were included as random factors in the LMM.

A secondary LMM analysis was conducted to examine the influence of measurement eccentricity upon the choroidal thickness changes with blind spot stimulation, using the data from the blue light and sham conditions on the entire study population (*n* = 20). The main effects of light (two levels: sham and blue light), measurement eccentricity (foveal, parafoveal and perifoveal), time, and refractive error and the three-way interaction of light by eccentricity by time and the four-way interaction of light by eccentricity by time by refractive error were included as fixed factors. The slope and intercept of individual participants were included as random factors. Bonferroni-corrected posthoc pairwise comparison tests were also conducted for any significant main effects or interactions. Changes in choroidal thickness and axial length are presented as the arithmetic mean ± standard error of the mean (SEM).

## Results

The demographic and ocular characteristics of the twenty participants enrolled in this study are displayed in Table 1.

**Table 1:** Comparison of the demographic and ocular characteristics of the emmetropic and myopic participants

	Emmetropes (*N* = 10)	Myopes (*N* = 10)	*P* value
Age (years)	28 ± 6	27 ± 6	0.64*
Gender (female, %)	30%	50%	0.65 †
Refractive error (D)	+ 0.02 ± 0.23	−3.01 ± 1.80	< 0.001 ‡
Axial length (mm)	23.90 ± 0.67	25.32 ± 1.25	0.005*
Subfoveal choroidal thickness (µm)	405 ± 98	304 ± 103	0.037*
Mean macular choroidal thickness (µm)	411 ± 95	305 ± 100	0.025*
Blind spot diameter (degrees)	5.68 ± 0.46	5.22 ± 0.55	0.06*

* Independent samples t-test, † Chi square test, ‡ Independent samples Mann-Whitney U test. Variations around the mean are indicated by standard deviation

### Changes in subfoveal choroidal thickness

**Fig. 3:**
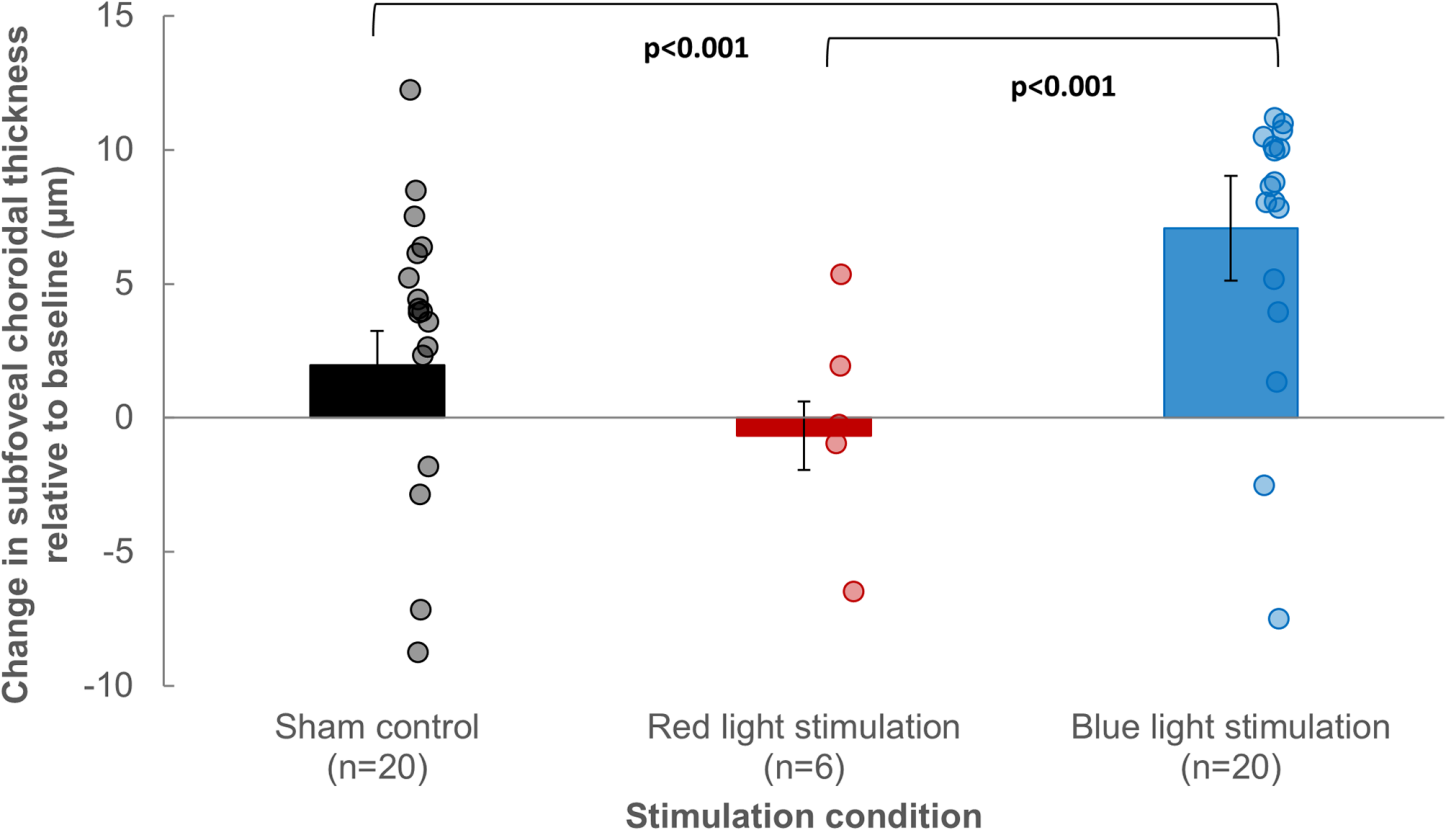
Changes in subfoveal choroidal thickness after a 60-minute period following stimulation of the blind spot with blue light, red light, or sham control for 1 min compared to the baseline measurements. Error bars indicate the standard error of the mean (SEM). Individual changes in subfoveal choroidal thickness after 60 min following 1-minute stimulation of the blind spot are represented by circular symbols. *p* < 0.001 for significant pairwise comparisons with Bonferroni correction between blind spot stimulation with blue light and red light, and with blue light and sham control

A statistically significant main effect of light was observed for changes in subfoveal choroidal thickness (F_2,362_=22.46, *p* < 0.001). An increase in subfoveal choroidal thickness was observed 60 min after blind spot stimulation with blue light (7 ± 1 μm, *n* = 20), which was significantly greater than the change observed following the sham control condition (2 ± 1 μm, *n* = 20) and following red light stimulation of the blind spot (−1 ± 2 μm, *n* = 6) using posthoc pairwise comparisons (both *p* < 0.001) (Fig. 3).

**Fig. 4:**
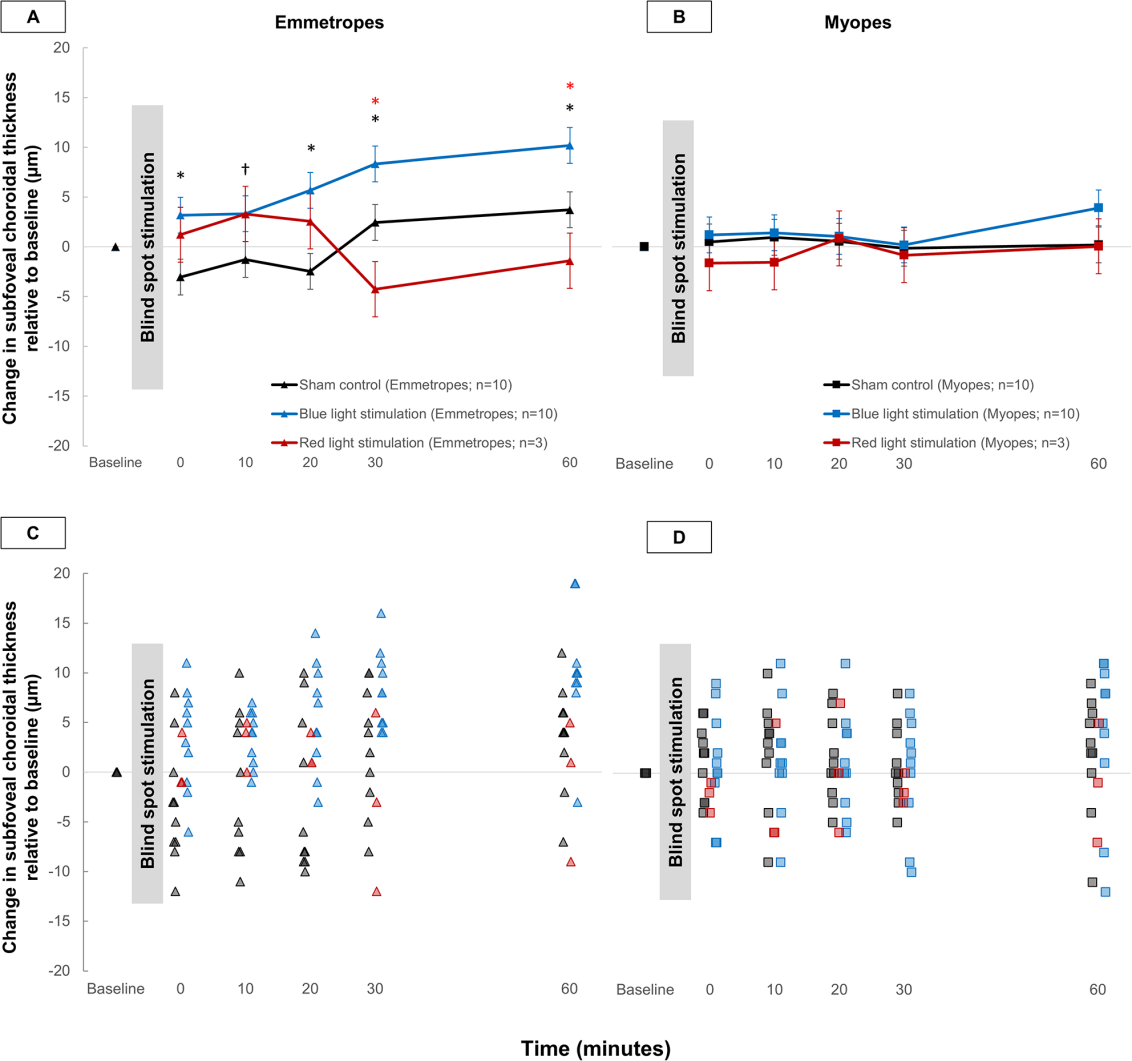
Time course of changes in subfoveal choroidal thickness over the 60-minute period following 1-minute stimulation of the blind spot with blue light compared to changes following sham control and red light stimulation in (**A**, **C**) emmetropes and (**B**, **D**) myopes. The error bars in (**A**) and (**B**) indicate the SEM. * *p* < 0.01, † *p* < 0.05 for significant pairwise comparisons with Bonferroni correction, indicated in black for choroidal thickness response to blue light stimulation of the blind spot versus sham control, and in red for choroidal thickness response to blue light stimulation of the blind spot versus red light stimulation

A statistically significant light by time by refractive error interaction was found (F_14,357_=3.17, *p* < 0.001), suggesting that the subfoveal choroidal response to blind spot stimulation with blue light varied with refractive error. In the emmetropes, a statistically significant increase in subfoveal choroidal thickness was found with blue light stimulation compared to the sham control immediately after (3 ± 2 μm vs. −3 ± 2 μm at 0 min, *p* < 0.01) and throughout the 60-minute poststimulation period (3 ± 2 μm vs. −1 ± 2 μm at 10 min, *p* < 0.05; 6 ± 2 μm vs. −2 ± 2 μm at 20 min, *p* < 0.001; 8 ± 2 μm vs. 2 ± 2 μm at 30 min, *p* < 0.01; 10 ± 2 μm vs. 4 ± 2 μm at 60 min, *p* < 0.01). The increase in subfoveal choroidal thickness with blue light stimulation of the blind spot in emmetropes was also significantly greater than the change in choroidal thickness with red light stimulation at 30 min (8 ± 2 μm vs. −4 ± 3 μm, *p* < 0.001) and 60 min (10 ± 2 μm vs. −1 ± 3 μm, *p* < 0.001) poststimulation. The subfoveal choroidal response to red light stimulation was not significantly different from the response to the sham control at any measurement time point (all *p* > 0.05). These findings suggest that the observed changes in choroidal thickness with blue light stimulation in emmetropes were associated with the wavelength of the stimulating light (Fig. 4A, C). In the myopic group, however, the changes in subfoveal choroidal thickness associated with blue light stimulation were not significantly different from the changes in choroidal thickness associated with the sham control or red light stimulation at any time point poststimulation (Fig. 4B, D).

**Fig. 5:**
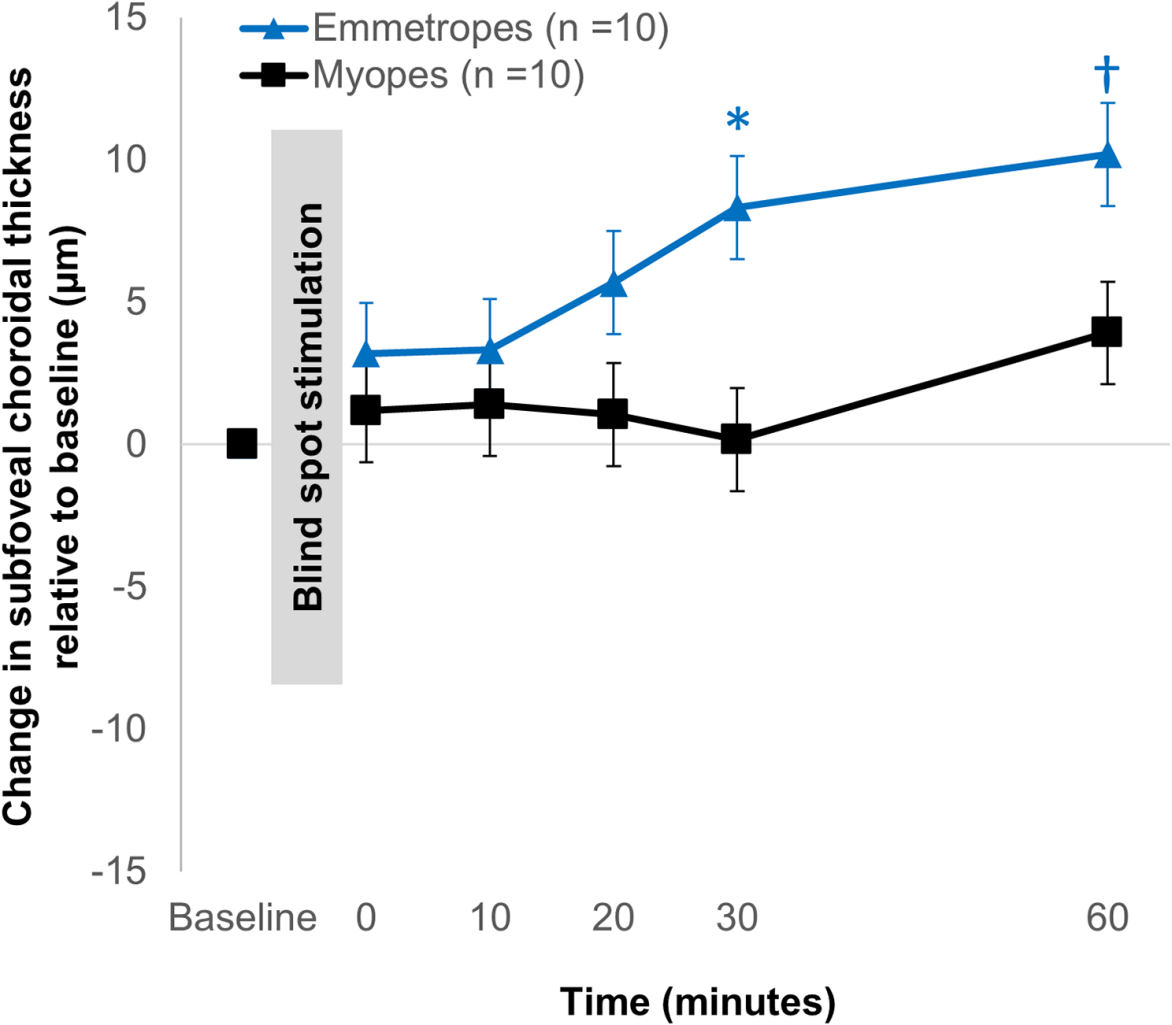
Time course of changes in subfoveal choroidal thickness over the 60-minute period following 1 min of stimulation of the blind spot with blue light compared to the baseline measurements in emmetropes (triangle symbols, *n* = 10) and myopes (square symbols, *n* = 10). Error bars indicate the standard error of the mean (SEM). * *p* < 0.01, † *p* < 0.05 with Bonferroni correction for comparison between the choroidal response to blue light stimulation of the blind spot in emmetropes and myopes

When the time course of changes in subfoveal choroidal thickness after blue light stimulation was compared between emmetropes and myopes, significantly greater subfoveal choroidal changes were observed in the emmetropes at 30 and 60 min after stimulation (Fig. 5).

### Changes in mean macular choroidal thickness

The analysis of changes in the mean macular choroidal thickness also revealed a statistically significant main effect of light (*p* < 0.001) and a statistically significant light by time by refractive error interaction (*p* < 0.05). However, the magnitude of change in the mean macular choroidal thickness with blind spot stimulation was generally less than the corresponding subfoveal change.

**Fig. 6:**
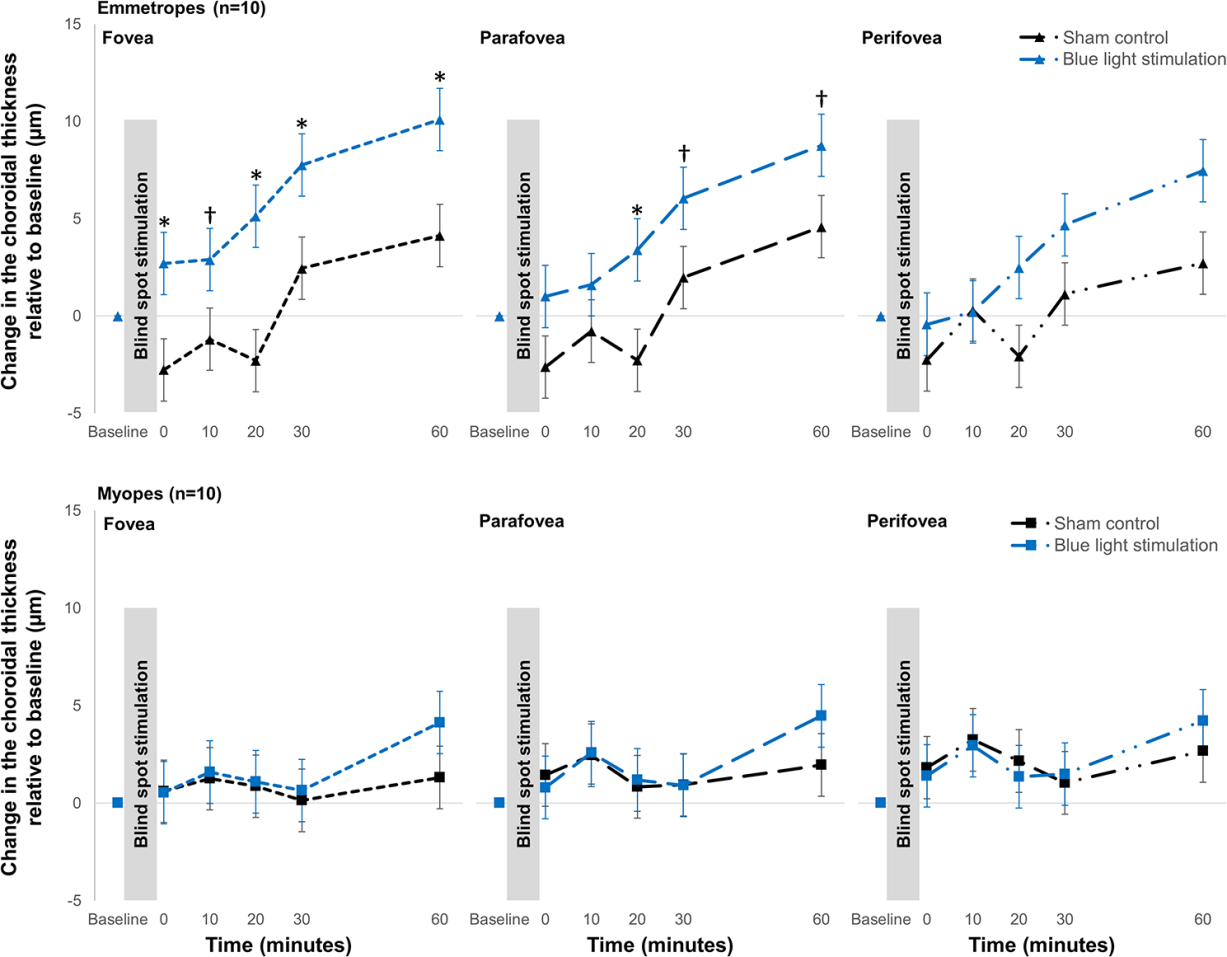
Time course of changes in choroidal thickness in the fovea (left graphs), parafovea (middle graphs), and perifovea (right graphs) over the 60-minute period following 1-minute stimulation of the blind spot with blue light or no light (sham control) compared to the baseline measurement in emmetropes (*n* = 10, top graphs) and myopes (*n* = 10, bottom graphs). Error bars indicate the standard error of the mean (SEM). * indicates paired comparisons *p* < 0.01 and † *p* < 0.05 with Bonferroni correction between the response to blue light stimulation of the blind spot and the sham control at the indicated time points

A statistically significant light by eccentricity by time by refractive error interaction was found (*p* < 0.001), suggesting that the differences in the mean macular choroidal thickness changes with blue light stimulation between emmetropes and myopes varied with eccentricity. In the emmetropes, the choroidal thickening observed with blue light stimulation was significantly greater than the corresponding changes in the sham control condition with no light across all the measured time points only in the foveal region. This refractive error-dependent choroidal response was attenuated in the parafoveal and perifoveal eccentricities (Fig. 6). Furthermore, compared with myopic individuals, emmetropes exhibited significantly greater choroidal thickening at 30 and 60 min post blue light stimulation in the foveal region (both *p* < 0.01), but these differences were diminished in parafoveal and perifoveal eccentricities.

### Changes in axial length of the eye

Relative to the baseline measurement, there was a trend for axial length to decrease by 6 ± 3 μm and 2 ± 3 μm at 60 min following 1 min of stimulation with blue light and sham control, respectively, and increase by 2 ± 5 μm at 60 min following 1 min of red light stimulation. However, these changes were not statistically significant with no significant main effect of light (F_2,25_=1.62, *p* = 0.21) or light by refractive error interaction (F_2,25_=1.56, *p* = 0.23) observed on axial length changes following blind spot stimulation.

## Discussion

This study provides evidence regarding the response of the human choroid to blue light stimulation of the blind spot which is likely to be driven by ipRGCs. A statistically significant thickening of the subfoveal choroid was found within one hour after a 1-minute blue light stimulation. The observed choroidal thickening after stimulation with flickering blue light was greater than the changes observed following two control conditions, namely, sham without any light stimulation and red light stimulation of the optic disc, suggesting that the wavelength of the stimulating light was the primary driver of the observed choroidal thickness changes. Qualitatively similar but smaller magnitude changes were also measured in the mean macular choroidal thickness compared to the subfoveal choroidal thickness.

The choroidal response to visual stimuli such as defocus (Delshad et al. 2020), accommodation (Hoseini-Yazdi et al. 2021; Woodman et al. 2011), and increased levels of non-flickering light (Chakraborty et al. 2022b; Thakur et al. 2021) is transient and decays within ∼ 20 to 30 min of stimulus offset. In the present study, the choroidal response to light stimulation of the blind spot was similar in magnitude to the responses to other visual stimuli applied in previous studies (Chakraborty et al. 2012, 2022b; Delshad et al. 2020; Hoseini-Yazdi et al. 2019, 2020, 2021; Lou and Ostrin 2020; Read et al. 2010, 2018; Thakur et al. 2021; Woodman et al. 2011). The change in choroidal thickness may be linked to an ipRGC dopamine signaling and its potential effects on choroidal vessels. There is evidence suggesting retrograde signaling in the ipRGCs in addition to forward signaling to the central nervous system, in which the axon collaterals of the ipRGCs influence the outer retinal layers through sustained firing of the dopaminergic amacrine cell (Joo et al. 2013; Prigge et al. 2016; Zhang et al. 2008) whereas a study on knock-out mice suggested that light regulation of retinal dopamine is independent of melanopsin phototransduction and originates primarily from rods and cones (Cameron et al. 2009). Recent studies have also provided further support for retrograde signaling of the ipRGCs in humans by demonstrating that the ipRGC-driven pathway in the retina is stimulated, and leads to activation of the retinal dopaminergic pathway following brief blind spot stimulation with blue light (Amorim-de-Sousa et al. 2021; Schilling et al. 2022). Therefore, it seems reasonable to assume that the observed choroidal response to blind spot stimulation with blue light is driven by retrograde signaling of the ipRGCs.

A recent study reported a decrease in choroidal thickness after 60 min of continuous full-field ocular exposure to red light (peak wavelength 630 nm, mean ± SD before 350.57 ± 89.68 μm and after 344.66 ± 88.77 μm) but not of continuous exposure to blue light (peak wavelength 456 nm, before 346.92 ± 86.68 μm and after 346.13 ± 88.23 μm). This difference in choroidal response was attributed to either an ipRGC-mediated retinal mechanism or a response to chromatic cues provided by the wavelength of light (Lou and Ostrin 2020). Short-term exposure to full-field blue light, but not red or green light, has also been found to negate the increase in axial length and decrease in choroidal thickness associated with exposure to hyperopic defocus in the human eye and leads to a relative shortening of axial length and thickening of the choroid (Thakur et al. 2021). This finding suggests that the regulation of axial length and choroidal thickness by full-field blue light may be driven by mechanisms that are not dependent on longitudinal chromatic aberrations, such as the S-cone excitatory input to the dopaminergic amacrine cells and/or the S-cone inhibitory input to the ipRGCs (Dacey and Packer 2003; Spitschan et al. 2014). The increase in choroidal thickness observed with blue light stimulation of the blind spot in the present study suggests that the choroid responds to intrinsic ipRGC-mediated retinal signaling mediated by melanopsin-expressing ipRGCs, which are sensitive to blue light (Hattar et al. 2002). This occurs in isolation from optical cues provided by blue light or S-cone driven signals due to the absence of conventional rod and cone photoreceptors at the optic disc.

One limitation of the study was that no continuous light was used. In a study using a flicker frequency of 10 Hz, it was shown that blue light stimulation of the blind spot elicited a stronger pupil response associated with melanopsin activation compared to red light (Adhikari et al. 2023). Therefore, it is likely that the 12 Hz blue light stimulus used in this study has also triggered the ipRGC-mediated signals at the blind spot.

Choroidal thickening to blind spot stimulation with blue light has been proven statistically only in emmetropic, but not myopic adults in this study. Recent studies have also revealed that the axial length of myopes do not respond to optical defocus as strongly as emmetropes (Swiatczak and Schaeffel 2021, 2022), although earlier studies do not support these findings (Chiang et al. 2015; Hoseini-Yazdi et al. 2020). Previous studies in both animals and humans have established a strong link between choroidal thickness and refractive development (Read et al. 2019; Troilo et al. 2019), with increases in choroidal thickness linked to mechanisms leading to emmetropia and decreases in choroidal thickness associated with mechanisms leading to myopia. Therefore, the differential change in choroidal thickness with blue light stimulation of the blind spot observed in emmetropes compared to myopes suggests the possibility that ipRGC-mediated signaling is involved in vision-dependent processes regulating the refractive state of the eye. Stronger ipRGC-mediated retinal signals might be associated with processes leading to emmetropia, and weaker signals might be associated with processes leading to myopia. A stronger ipRGC-mediated pupillary response in emmetropes than in myopes has also been demonstrated, with a greater post illumination pupillary response following repeated exposure of the pupil to blue light found in non-myopic young adults than in myopic young adults (Mutti et al. 2020).

In contrast, a recent study revealed a significant increase in retinal electrophysiological responses from the inner plexiform layer (amacrine and bipolar cells) and from the retinal ganglion cells of myopic, but not emmetropic, eyes 20 min after 1 min stimulation of the blind spot with blue light (Amorim-de-Sousa et al. 2021). However, these retinal changes were examined under photopic illumination, unlike the mesopic conditions in the present study, suggesting that a possible interaction between intrinsic and extrinsic cone-mediated ipRGC signaling pathways may influence the responses to blue light stimulation of the blind spot in different refractive groups. Alternatively, ipRGC-mediated retinal signaling may remain intact in both myopes and emmetropes, with the differences in choroidal thickness changes in response to ipRGC signaling observed in the present study explained by differences in downstream signaling in the dopaminergic pathway of myopic eyes, such as in the retinal pigment epithelium (Dearry et al. 1991; Rymer and Wildsoet 2005; Zhang et al. 2009) and/or the choroid (Hoseini-Yazdi et al. 2021; Nickla et al. 2010; Nickla and Wallman 2010; Reitsamer et al. 2004). Given that myopia typically develops during childhood and that only young adults with established myopia were included in this study, it has yet to be determined whether the smaller ipRGC-mediated changes in choroidal thickness observed in myopes contribute to the cause or are a consequence of myopic eye growth.

There was also eccentricity-dependent variation in the choroidal response to ipRGC-mediated signaling in emmetropes compared to myopes, with greater changes observed in the foveal region than in the parafoveal and perifoveal regions (Fig. 6). This greater foveal choroidal thickness in response to ipRGC-mediated signals from outside the fovea may be explained by the greater density of the ipRGC in the central 2 mm region of the inner retina (Nasir-Ahmad et al. 2019). In the central region of the retina, the choroidal thickening may be more responsive to ipRGC-mediated signaling via the retrograde dopaminergic pathway as shown in rodents and primates (Prigge et al. 2016; Joo et al. 2013).

Short-term changes in choroidal thickness are typically inversely associated with changes in axial length. An increase in choroidal thickness causes forward movement of the retinal pigment epithelium (RPE) and shortening of the axial length since axial length is measured from the anterior corneal surface to the RPE. Consistent with the ipRGC-mediated increase in choroidal thickness, the results also showed a trend for axial length to decrease with blue light stimulation compared to that of the sham control and red light stimulation of the blind spot. Although these changes were similar in magnitude to the changes in choroidal thickness, these changes in axial length were not statistically significant. The spatial resolution of the Lenstar optical biometer is ∼ 10 μm (https://haag-streit.com/2Products/Specialitydiagnostics/Biometry/Lenstar900/Instructionsforuse/1500_7220055_04150_IFU_Lenstar_LS_900_01_en_web.pdf), whereas the spatial resolution of the Spectralis OCT is 3.9 μm (https://www.heidelbergengineering.com/download.php?https://media.heidelbergengineering.com/uploads/Products-Downloads/200279-002-INT-AE18_SPECTRALIS-Technical-Data-Sheet_EN.pdf). Although state of the art was used for the measurement of choroidal thickness and axial length of the eye, these technical shortcomings of the study allow clear conclusions on a physiological response for values above these spatial resolutions, whereas low values could only be a statistical effect. Especially the lower precision and greater short-term variability in axial length compared to choroidal thickness measurements may underlie the greater variability in the axial length response to blind spot stimulation with light and the non-significant differences. Since the sample size of the study was only calculated for choroidal thickness, it is possible to find statistically significant effects with a more appropriate sample size optimized for axial length.

## Conclusions

In summary, a significant thickening of the human choroid was observed within one hour after stimulation of the blind spot with blue light for one minute. The magnitude of choroidal thickening 60 min after blue light stimulation was statistically significant and above the spatial resolution of OCT assessment in individuals with emmetropia. This effect was more prominent in the foveal region than in the extra-foveal areas of the macula.

This response is likely related to the ipRGC-mediated retinal signaling pathway, as rods or cones are not present in the optic disc. Thus, choroidal thickness could serve as a short-term, noninvasive, objective surrogate marker of the intrinsic activity of the ipRGCs in the human eye. Given that the short-term increase in choroidal thickness is known to be a biomarker of longer-term ocular mechanisms that lead to emmetropia, the observed choroidal thickening after blue light stimulation in this study may have implications for the control of eye growth.

## Data Availability

The datasets used and/or analyzed during the current study are available from the corresponding author upon reasonable request.

## Abbreviations

AxL: Axial length
ipRGC: Intrinsically photosensitive retinal ganglion cell
OCT: Optical coherence tomography
SD: Standard deviation
VR: Virtual reality
